# A Tutorial for Isolating, Characterizing, and Inducing Presenescence in Human Periodontal Ligament and Dental Pulp Stem Cells

**DOI:** 10.1002/cpz1.70370

**Published:** 2026-04-07

**Authors:** Kamila Sauer Veiga Leme, Márjorie de Assis Golim, Aline Márcia Marques Braz, Elenice Deffune, Daisy Maria Fávero Salvadori

**Affiliations:** ^1^ São Paulo State University (Unesp) School of Medicine Botucatu Brazil; ^2^ University of Detroit Mercy Dental School Michigan

**Keywords:** cell isolation, cell characterization, cell presenescence, human dental pulp stem cells, human periodontal ligament stem cells, stem cells

## Abstract

For several years, scientists have focused on studying mesenchymal stem cells because of their ability for self‐regeneration, their potential for differentiation into multiple lineages (e.g., osteogenic, chondrogenic, and adipogenic cells), and their low immunogenicity and remarkable capacity to modulate the immune system. The importance of these cells ranges from preserving tissue health and repairing injured tissues in their nearby areas, to their use in scientific investigation for the treatment of neurodegenerative, autoimmune, and cardiovascular diseases. Despite the availability of various tissue sources, such as bone marrow and adipose tissue, their collection and handling may not always be easily achievable. However, dental tissues, such as the pulp and periodontal ligament, are relatively accessible supplies that do not require complex or stressful interventions for the patient. Here, we provide a detailed description of each step involved in the isolation and characterization of mesenchymal stem cells from the pulp and periodontal ligament using monoclonal antibodies ensuring a high level of effectiveness. In addition, we also describe a technique to generate the cellular presenescence state. © 2026 The Author(s). *Current Protocols* published by Wiley Periodicals LLC.

**Basic Protocol**: Isolation of human periodontal ligament and dental pulp stem cells

**Support Protocol 1**: Characterization of human periodontal ligament and dental pulp stem cells

**Support Protocol 2**: Induction of presenescence of human periodontal ligament and dental pulp stem cells

## INTRODUCTION

Mesenchymal stem cells (MSCs) are a type of cells that can be found in bone marrow, adipose, and other tissues, such as dental tissues like human dental pulp stem cells (HDPSCs) and human periodontal ligament stem cells (HPLSCs). These cells can be differentiated into various other lineages (osteogenic, chondrogenic, adipogenic) when cultured in the laboratory. MSCs have low immunogenicity and strong immunomodulation potential (Egusa et al., 2012; Horwitz et al., 2005). Despite limited availability in adult tissues, these cells have the capacity to undergo self‐renewal and differentiation to maintain the health of tissues while promoting the recovery of injured tissues (Egusa et al., 2012). Furthermore, HDPSCs can also differentiate into odontoblasts (specialized osteoblasts) and into nerve cells (astrocytes, glial cells, and oligodendrocytes). These cells come from the embryonic ectomesodermal tissue that gives rise to neural crest (Lampiasi, 2023). Numerous investigations have demonstrated that these cells exhibit capacities that extend beyond dentin regeneration in teeth (Gronthos et al., 2000). They also have a positive impact on neurodegenerative diseases such as Alzheimer's disease, Parkinson's disease, vascular dementia, among others (Apel et al., 2009; Candelise et al., 2023; X. M. Zhang et al., 2021).

In contrast to HDPSCs, the population of stem cells obtained from the periodontal ligament primarily consists of mesenchymal stem cells with a small fraction of neural crest stem cells. However, HPLSCs additionally can differentiate into neurogenic, cardiomyogenic, chondrogenic, and osteogenic lineages, as well as having fibrinogenic and cementogenic potential (Huang et al., 2009; J. Liu et al., 2020). The significance of these cells in maintaining and stabilizing the periodontal ligament, as well as their role in cellular renewal and functionality following cementum injuries, is extensively documented. Nevertheless, in recent years the potential for differentiation and paracrine effects on neural regeneration mechanisms have also been explored (Calabrese, 2021; Mohebichamkhorami et al., 2022; Ng et al., 2015).

Literature has described different methods for isolating and characterizing cells, which exhibit significant variation in several aspects, including enzymatic digestion (Chen et al., 2020; Cui et al., 2014), the explant method (Hilkens et al., 2013; Patil et al., 2018), and types and times of collagenase for tissue breakdown (Du et al., 2016; Ehlinger et al., 2021). The composition of the supplemented medium, which includes the use of fetal bovine serum and/or growth factors is also considered (di Vito et al., 2020; Kawase‐Koga et al., 2020; Zheng et al., 2018). Furthermore, the studies examine the use of specific monoclonal antibodies (positive and negative types) for mesenchymal stem cells (T. Liu et al. 2020; S. Zhang et al., 2019), along with other characterization methods, such as clonogenic formation (Yang et al., 2015) and induction of cell differentiation (Liang et al., 2022; Marconi et al., 2021). Despite the complexity of cellular processes, the technique detailed here utilizes materials routinely found in cell culture laboratories and requires fewer reagents compared to other approaches. It is a rapid and straight forward methodology that can be easily replicated and reliable.

The development of an effective cell isolation and characterization approach is crucial, especially in the context of stem cells. However, they may exhibit sensitivity to premature senescence in pathological conditions induced by oxidative stress (Zhou et al., 2015). During the stage of presenescence, cells are at a critical point when they can undergo significant cellular repair transition into senescence (Akagi et al., 2023). Therefore, the protocols described here provide a rapid and high‐effective method for investigating stem cells in a presenescence stage.

*NOTE*: Appropriate informed consent is necessary for obtaining and use of human study material.

## ISOLATION OF HUMAN PERIODONTAL LIGAMENT AND DENTAL PULP STEM CELLS

An extensive clinical examination of the teeth to be used is crucial, ensuring they are free from cavities, restorations, periodontal disease, and any form of trauma, such as clenching or bruxism. Impacted or unerupted teeth are desirable since they have not been exposed to the external environment. The teeth selected for extraction are often the third molars, which must be removed for orthodontic treatment purposes. To provide an adequate experimental design, it is recommended to collect tooth specimens from a total of six patients (see Supporting Information, Table S1). Subsequently, select three of those that give the most precise cellular characterization, based on the antibodies employed, for conducting the investigation. Thus, genetic diversity is properly addressed.

*NOTE*: Completing an anamnesis form and obtaining the patient's consent are crucial tasks (see Supporting Information, Ethical approval). Patients who smoke, consume alcohol, or have systemic disorders are not eligible.

*NOTE*: This methodology is suitable to both the pulp and the periodontal ligament. When performing simultaneous isolation of both tissues, it is important to separate the samples to prevent cross‐contamination even if they come from the same patient.

### Materials (also see Table 1)

SCM20% (see recipe)
Phosphate‐buffered saline (PBS) (Gibco, cat. no. 20012027)
3 mg/ml collagenase type I solution (see recipe)
0.05% Trypsin‐EDTA (Gibco, cat. no. 25300062)
0.4% Trypan blue solution (Gibco, cat. no. 15250061)
SCM10% (see recipe)

250‐ml glass bottle (Prolab, cat. no. 21801145)
5‐ml serological pipette, sterile (SPL Life Sciences, cat. no. 91005)
10‐ml serological pipette, sterile (SPL Life Sciences, cat. no. 91010)
25‐ml serological pipette, sterile (SPL Life Sciences, cat. no. 91025)
1.5‐ml microcentrifuge tubes (Kasvi, cat. no. K6‐0150)
Sterile specimen collection cup, 80‐ml (JProlab)
Dental clinic tweezers (Quinelato, cat. no. QC 115‐15)
Scalpel blade, no. 23 (Advantive)
Wood chisel, 8‐mm (Stanley)
Dental mallet (Quinelato, cat. no. QD.992.16)
Dental spoon excavator, no. 11.5 (ICE)
Styrofoam box, 3‐L (Placterm) with ice
Gauze pads, 7.5 × 7.5–cm (Ultracotton)
5‐ml syringe (Becton Dickinson, cat. no. 309646)
24G, 0.75‐in. hypodermic needle (Becton Dickinson, cat. no. 302023)
0.22‐micron syringe filter (Kasvi, cat. no. K18‐230)
Iris scissors curved (Quinelato, cat. no. Qt82311)
70‐µm cell strainer (Corning, cat. no. 431751)
50‐ml Falcon tube (Falcon, cat. no. 352070)
15‐ml Falcon tube (Falcon, cat. no. 352096)
35 × 10–mm cell culture dish (Corning, cat. no. 430165)
1000‐µl filter tips (Axygen, cat. nos. AXYTF1000RS)
T‐25 cm² cell culture flask (Corning, cat. no. 353136)
T‐75 cm² cell culture flask (Corning, cat. no. 430639)
Container for discards

**Table 1:** Equipment Required at Each Cell Stage in Basic Protocol and Support Protocols 1 and 2

Cell stage	Dental tissues collection and storage (periodontal ligament and pulp) (Basic Protocol)	Dental cells isolation (Basic Protocol)	Cellular establishment and expansion (Basic Protocol)	Cell characterization (Support Protocol 1)	Presenescence induction (Support Protocol 2)
*Required equipment*:					
Laminar flow cabinet	✓	✓	✓	✓	✓
100‐ to 1000‐µl micropipette	✓	✓	✓	✓	✓
Analytical balance		✓			
Refrigerator	✓				
Water bath	✓	✓	✓	✓	✓
Vortex		✓		✓	
Centrifuge (5702 R, Eppendorf)		✓	✓	✓	
CO_2_ incubator (Forma Series II water jacketed CO_2_ incubator, Thermo Fisher Scientific)		✓	✓		✓
Inverted microscope			✓	✓	✓
Neubauer chamber			✓	✓	
Flow cytometer (BD FACSCalibur 4 colors, Becton Dickinson)				✓	

#### Dental tissues collection and storage (periodontal ligament and pulp)

All equipment used in the “Dental tissues collection and storage (periodontal ligament and pulp)” stage will be necessary for the preparation, storage, and thawing of the supplemented culture medium that will be used to maintain the collected dental tissues.

1 In a cell culture room, prepare SCM20% solution in a 250‐ml glass bottle using serological pipettes inside the laminar flow hood. Take 1 ml of this solution and transfer it into a microcentrifuge tube for each tissue sample to be collected. Additionally, aliquot 15 ml PBS into a sterile specimen collection cup.
2 In the dental clinic, immediately after tooth extraction, hold the element with tweezers and wash in PBS to remove excess blood.
3 To collect the periodontal ligament, scrape the entire medial third of the tooth roots with a scalpel blade and place it inside the microtube with SCM20%.
4 To collect the pulp, hold the tooth firmly, resting the crown on the bench with the chisel resting between the roots, in the furcation area and hitting the chisel head with the dental mallet.
5 Collect the pulp with a curette (dental spoon excavator) and place the tissue in the other microtube with SCM20%.
6 Transport dental tissue in styrofaom box with ice. Proceed to cell isolation.
  It is advisable to wear protective glasses while breaking the tooth root because there is a possibility of dislodging enamel fragments.
  Using gauze pads to provide support to the tooth´s crown aids in the case of root fracture.
  Dental tissues, pulp and ligament, can also be stored in the SCM20% at 4°C overnight for later isolation.

#### Dental cell isolation

7 Prepare type I collagenase solution. Use a syringe and hypodermic needle to collect all collegenase solution from microtube after mixing and use a 0.22‐micron syringe filter to filter the solution into a new microtube.
8 Remove SCM20% from the microtubes containing the tissues and wash twice with PBS.
9 After removing as much of the PBS from the microtubes as possible, add the freshly prepared and filtered type I collagenase solution.
10 Carefully cut the tissues into very small pieces with scissors inside the microtubes, avoiding extravasation of the collagenase solution.
11 Place the microtubes in the water bath at 37°C for 1 hr. During this period, gently mix the collagenase solution with the tissues by inverting the microtubes every 15 min.
12 After 1 hr of collagenase activity on the tissues, filter the solution through a 70‐µm cell strainer into a 50‐ml Falcon tube and add 1 ml of SCM20% to neutralize the collagenase.
13 Centrifuge the sample in a 15‐ml Falcon tube for 10 min at 200 × *g*, 25°C.
14 Remove the supernatant and add 300 µl SCM20% to the pellet. Mix carefully and transfer to a 35 × 10–mm cell culture dish.
15 Add 3 ml of SCM20% to the dish and move it to the incubator at 37°C with 5% CO_2_.
  The amount of tissue obtained during collection will be very small, therefore the steps of removing SCM20% and PBS must be performed very carefully. It is crucial to allow the tissue to settle at the bottom of the microtube and to gently remove the liquids using a tip that is in touch with the surface, avoiding contact with the bottom of the microtube.
  As collagenase interacts with tissues, it is typical to observe the disintegration of the tissues and the formation of a pellet‐like particle.
  It is imperative to maintain the temperature at 37°C and 5% CO_2_ in the incubator without any changes at this moment.

#### Cellular establishment and expansion

##### Third and sixth day after cell isolation

16 Make a partial change of SCM20%.
17 Remove 1.5 ml of SCM20% and add 1.5 ml of fresh SCM20%.
18 Observe under the microscope whether there are adhered cells and whether there is no contamination.
19 Return the dish to the incubator.

###### Ninth day after cell isolation

20 Wash the dish with PBS.
21 Remove all the medium with the tip touching the edge of the dish.
22 Add 1 ml PBS, uniformly along the edge of the plate, gently agitate and remove the PBS.
23 Add 3 ml of the SCM20% solution and place the dish back into the incubator.

###### Twelfth day after cell isolation

24 Wash the cells with PBS.
25 Add 500 µl trypsin, wait ∼2 min while observing the cells detach under a microscope and add 500 µl of SCM20%.
26 Centrifuge for 10 min at 200 × *g*, 25°C.
27 Remove the supernatant and place the pellet in a T‐25 cm² flask, adding 5 ml SCM20%.
28 Place the T‐25 cm² flask in the incubator at 37°C with 5% CO_2_.

###### P0 to P1 passage (expansion)

29 When reaching 80% cell confluence in the T‐25 cm^2^ flask, expand the cells.
30 Wash cells with 2 ml PBS.
31 Add 1 ml trypsin, spread well, wait ∼2 min, and gently tap the flask to release the cells.
32 Add 1 ml SCM20% and centrifuge for 10 min at 200 × *g*, 25°C.
33 Remove the supernatant and homogenize the pellet in 1 ml SCM20%.
34 Collect 50 µl of the cell suspension, add 50 µl trypan blue, homogenize, and take 10 µl for cell counting.
35 Plate the cells in T‐75 cm² flasks with 15 ml SCM10% at a maximum concentration of 5000 cells/cm².
36 Place the T‐75 cm² flasks in the incubator at 37°C with 5% CO_2_.
  Partial and complete replacements of the SCM20% must be made very carefully with the tip on the surface of the solution and at the edge of the dish.
  It is advisable to perform partial replacement of SCM20% on two occasions, specifically on the third and sixth days after isolation, or until small groups of cells becomes visible.
  It is crucial to partially exchange the SCM20% in order to maintain the growth factors.
  It is expected to see a significant number of red blood cells and cellular debris until the first trypsinization and particularly until the first wash with PBS. Normally, recently detached cells hidden by this material.
  Cells can also be frozen from the first passage instead of being expanded.
  When the pulp and ligament are isolated simultaneously, even from the same patient, there may be differences in the growth and development of the two cultures. Usually, the first cells to grow after isolation are those derived from the ligament. These cells exhibit more resistance to variations in temperature, pressure, and composition of the culture media. Therefore, the duration of partial or complete SCM20% exchange, as well as cell trypsinization, may vary between both.

## Support Protocol 1 CHARACTERIZATION OF HUMAN PERIODONTAL LIGAMENT AND DENTAL PULP STEM CELLS

Antibodies must be validated and titrated in order to identify the appropriate volume for use: one that does not present non‐specific reactions (cross‐reactions), and, at the same time, which is sufficient to mark the positive cells. It is essential to test the concentrations that will be utilized in the characterization. In this protocol, the volumes used were 1 µl for each isotypic control, 5 µl for CD105 FITC, 1 µl for CD34 PE, 0.1 µl CD45 PercP, and 2.5 µl of CD90 APC (see Supporting Information, Table S1). The software used was Cell Quest Pro and Flow Jo.

### Materials (also see Table 1)

Plated cells from Basic Protocol
PBS (Gibco, cat. no. 20012027)
Monoclonal antibodies:
  CD105, FITC (Invitrogen, cat. no. MA1‐1959‐4)
  CD34, PE (Invitrogen, cat. no. 12‐0349‐41)
  CD45, PerCP‐eFluor (Invitrogen, cat. no. 46‐0459‐41)
  CD90, APC‐eFluor 780 (Invitrogen, cat. no. 47‐0909‐41)
Isotype control mouse antibodies:
  IgG2a, k, FITC (BD Pharmingen, cat. no. 553456)
  IgG1, k, PE (BD Pharmingen, cat. no. 550617)
  IgG1, k, PerCP (BD Pharmingen, cat. no. 550672)
  IgA, k, APC (BD Pharmingen, cat. no. 562140 )
ISOTON II diluent (Beckman Coulter, cat. no. 8546719)

1.5‐ml microcentrifuge tubes (Kasvi, cat. no. K6‐0150)
10‐ to 1000‐µl filter tips (Axygen, cat. nos. AXYTF300RS, AXYTF100RS, and AXYTF1000RS)
12 × 75–mm, 5‐ml Falcon polystyrene tube (Falcon, cat. no. 352235)

1 Wash, trypsinize, and count the plated cells (as outlined in Basic Protocol, steps 30 to 34).
2 Transfer ∼1 × 10⁶ cells to a microtube with 1 ml PBS for characterization.
3 Homogenize the cells and transfer 100 µl of the suspension into a tube for isotypic control, another 100 µl into a tube for autofluorescence control, and an additional 100 µl into a tube for antibodies.
4 Add the titrated volumes of antibodies to the cells.
5 Vortex the tubes and incubate for 30 min in the dark and at room temperature.
6 Add 500 µl ISOTON II, vortex, and centrifuge for 10 min at 400 × *g*, 25°C.
7 Remove the supernatant and resuspend in 500 µl ISOTON II.
8 Obtain the measurement on the flow cytometer, with a count of 30,000 events per tube.
  It is possible to simultaneously utilize antibodies labeled with distinct fluorochromes in the flow cytometer, as they can be detected through separate channels.
  Although there are several positive and negative surface molecules, here, we select two positive markers (CD105 and CD90) and two negative markers (CD34 and CD45) in the characterization of the mesenchymal stem cell immunophenotype.
  In the context of cellular immunophenotyping, isotypic control refers to a tube containing antibodies that do not react with the cell being evaluated, but which are specific for other species. However, these must be labeled with the same fluorochromes and belong to the same class and subclass of antibodies as those used in the test tube to evaluate the cellular immunophenotype to ensure optimal background fluorescence in the tube, even if it is not linked to the cell.
  It is important to note that the autofluorescence control is designed to assess the cellular autofluorescence of each sample, which must be excluded from the subsequent analysis. The tube must be subjected to the complete labeling protocol, including all requisite centrifugations and washes. It is imperative that monoclonal antibodies not be pipetted.
  Characterization of mesenchymal stem cells should be performed in the second to third passage, as before that, the cell population will exhibit significant heterogeneity and will not have the necessary characteristics to be classified as mesenchymal stem cells.

## Support Protocol 2 INDUCTION OF PRESENESCENCE OF HUMAN PERIODONTAL LIGAMENT AND DENTAL PULP STEM CELLS

This protocol was developed to verify the ability of these cells to keep and to compare their morphology and structure while stem cells in front of the induction of presenecente stage. This protocol is optional. It can be done when it is important to evaluate the integrity and response of these cells together with different materials, or in late passage, as a positive control. Besides that, it shows that, although the stem cells can be regenerated, in a cell culture, the presnescence stage can be reached.

### Materials (also see Table 1)

Stem cells from Basic Protocol after Support Protocol 1
SCM10% (see recipe)
150 µM hydrogen peroxide solution in SCM10% (see recipe)
PBS (Gibco, cat. no. 20012027)

35 × 10–mm culture dish (Corning, cat. no. 430165)
15‐ml Falcon tube (Falcon, cat. no. 352096)
10‐ to 1000‐µl filter tips (Axygen, cat. nos. AXYTF300RS, AXYTF100RS, and AXYTF1000RS)
Container for discards

1 Culture ∼1 × 10⁵ cells in two 35 × 10–mm dishes for 24 hr.
2 Prepare the intermediate solution and 150 µM hydrogen peroxide solution in a 15‐ml Falcon tube using filter tips (see Reagents and Solutions for details).
3 Remove the SCM10% from the dish that will receive the treatment and add 2 ml of the 150 µM H_2_O_2_ solution.
4 Incubate for 2 hr in an incubator at 37°C and 5% CO_2_.
5 After 2 hr, wash the two dishes, one containing untreated cells and the other with treated cells, using PBS. Subsequently, add 2 ml SCM10% into each dish.
6 Place the dishes in the incubator at 37°C with 5% CO_2_ for 24 hr.
  Ensure that two dishes are always prepared, with one serving as the negative control containing untreated cells.
  Higher concentrations of H_2_O_2_ also induce senescence, but there is considerable cell death.
  When cells reach confluence, they also undergo senescence. Therefore, it is crucial to carefully consider the experimental design, the number of cells plated, and the duration of the experiment.

## REAGENTS AND SOLUTIONS

### Collagenase type I solution, 3 mg/ml

Used for dental cell isolation. Before starting, weigh collagenase type I (3 mg/ml). This step can be done ahead of time. Store up to 6 months at 4°C. Suggestion: collagenase can be weighed inside the microtube where the solution will be made and use a microtube for each cell type. To prepare 1500 µl of the solution, add 1300 µl Dulbecco's modified Eagle medium (DMEM) (Gibco, cat. no. 11885084) to the microtube with 0.0045 g of collagenase type I (Gibco, cat. no. 17100017), vortex for 3 min, add 200 µl DMEM, mix by inversion, and filter through 0.22‐µm filter (Kasvi, cat. no. K18‐230). Place the filtered solution in a new microtube.

This solution must be prepared at the time of cell isolation.

This solution cannot contain FBS as FBS inhibits the action of collagenase.

The optimal temperature of solution is 37°C. The water bath and the medium must be at 37°C.

To compensate for the loss of solution during filtering, a volume of 1.5 ml needs to be prepared for a desired volume of 1 ml.

This volume of working solution is sufficient for tissue isolation of 2 to 3 teeth, pulp or periodontal ligament.

### Hydrogen peroxide solution in SCM10%, 150 µM

Used for presenescence induction. To prepare 2200 µl of 150 µM H_2_O_2_ solution, make an intermediate solution by adding 1.7 µl hydrogen peroxide (H_2_O_2_) (Sigma Aldrich, cat. no. 386790‐M) to 998.3 µl SCM10% (see recipe), mix by inversion, add 22 µl of the intermediate solution to 2.178 µl SCM10% (see recipe), and mix gently by inversion several times.

This solution must be freshly prepared.

Pay attention to possible H_2_O_2_ droplets on the outside of the tip, these droplets increase the molarity of the solution.

This preparation volume is for a 35 × 10–mm cell culture dish or one well of a 6‐well culture plate.

### SCM10%

Supplemented culture medium 10% SFB (SCM10%), used for cellular establishment and expansion. To prepare 100 ml of SCM10%, mix 89 ml DMEM (Gibco, cat. no. 11885084), 10 ml sterile inactivated fetal bovine serum (FBS) (Cultilab, cat. no. F063) and 1 ml penicillin‐streptomycin (P/S), 10,000 U/ml (Gibco, cat. no. 15140122). Shake the bottle to mix well. Store up to 1 week at 4°C.

The SCM10% will be used from the first passage which will be counted from the moment the cells are in the T‐25 cm² cell culture flask and go to the T‐75 cm² (Egusa et al., 2012) cell culture flasks.

The SCM10% will also be used for characterization and induction of cellular presenescence.

### SCM20%

Supplemented culture medium 20% SFB (SCM20%), used for dental tissue collection and storage (periodontal ligament and pulp). To prepare 50 ml of SCM20%, mix 39.5 ml DMEM (Gibco, cat. no. 11885084), 10 ml FBS (Cultilab, cat. no. F063) and 500 µl P/S (Gibco, cat. no. 15140122). Shake the bottle to mix well. Store up to 1 week at 4°C.

At this time, the SCM20% can be used cold to maintain dental tissues until cell isolation.

The volume of SCM20% will be for this step, to dental cell isolation and cellular establishment.

## COMMENTARY

### Troubleshooting

Adjustments may be necessary when certain problems occur, such as those listed in Table 2.

**Table 2:** Troubleshooting Guide for Potential Problems Encountered While Executing These Protocols

Stage	Problem	Suggestions/comments
Dental tissues collection	Difficulties in obtaining the tooth and collecting the pulp	When the roots are connected and it is difficult to reach the point that they separate, a groove can be made with the high‐speed dental handpiece and a small diamond ball burr
Dental cell isolation	Failure to obtain cells	Check type, proportion, and preparation of collagenase Check the collagenase activity time and water bath temperature
Cellular establishment and expansion	Culture contamination	Prepare small amounts of supplemented medium at a time and always discard the tip after each usage
		If you are expanding both pulp and ligament, it is advisable to work on each one separately
	Difficulty in cell expansion	Cell concentration (cells/cm²) lower than ideal in plating
		Incubator with unregulated temperature and CO_2_ percentage
		Acidified supplemented medium
Cell characterization	Difficulty in cell characterization	Cell content <1×10⁵ cells
		Use of very early passage cells (first/second passage)
		Use of incorrect antibody concentration and timing
Presenescence induction	Difficulties in establishing presenescence induction	Wrong H_2_O_2_ concentration/dilution
		Cell incubation time is shorter than 24 hr after treatment

### Results and Discussion

The described method demonstrates two out of the three minimum criteria cited by the International Society of Cellular Therapy to define human MSC: must be plastic‐adherent when maintained in standard cultural conditions; must express CD105, CD73, and CD90; and lack expression of surface molecules CD45, CD34, CD14 or CD11b, CD79‐Alpha or CD19 and HLA‐DR (Dominici et al., 2006). Additionally, it presents a technique for inducing the state of presensescence. The quality of the results achieved depends upon the detail of how the procedure was performed: from patient selection, clinical dental assessment and filling out the anamnesis form, to the careful handling of the cells.

**Figure 1:**
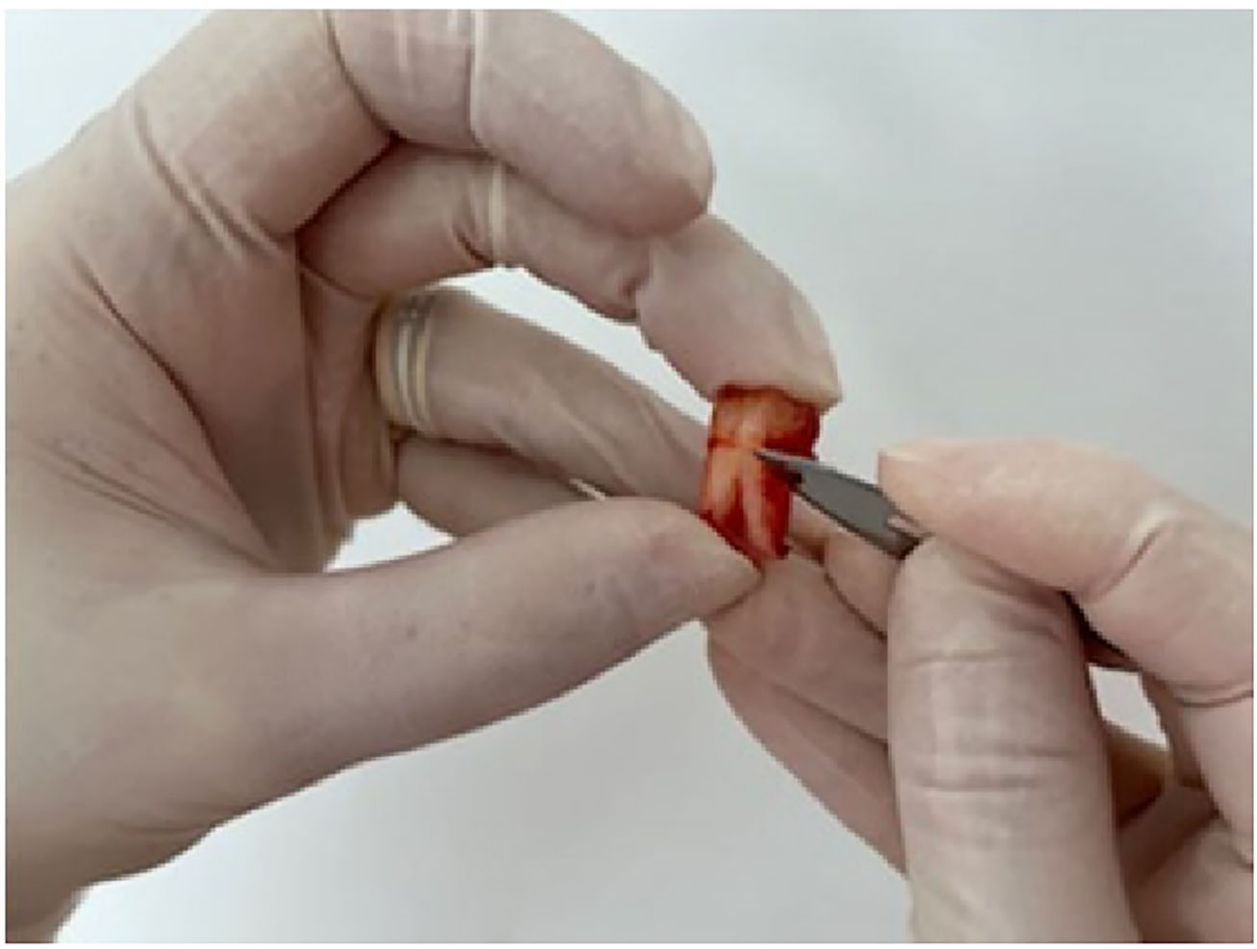
Harvesting the periodontal ligament. Periodontal ligament tissue scraped off the tooth root.

**Figure 2:**
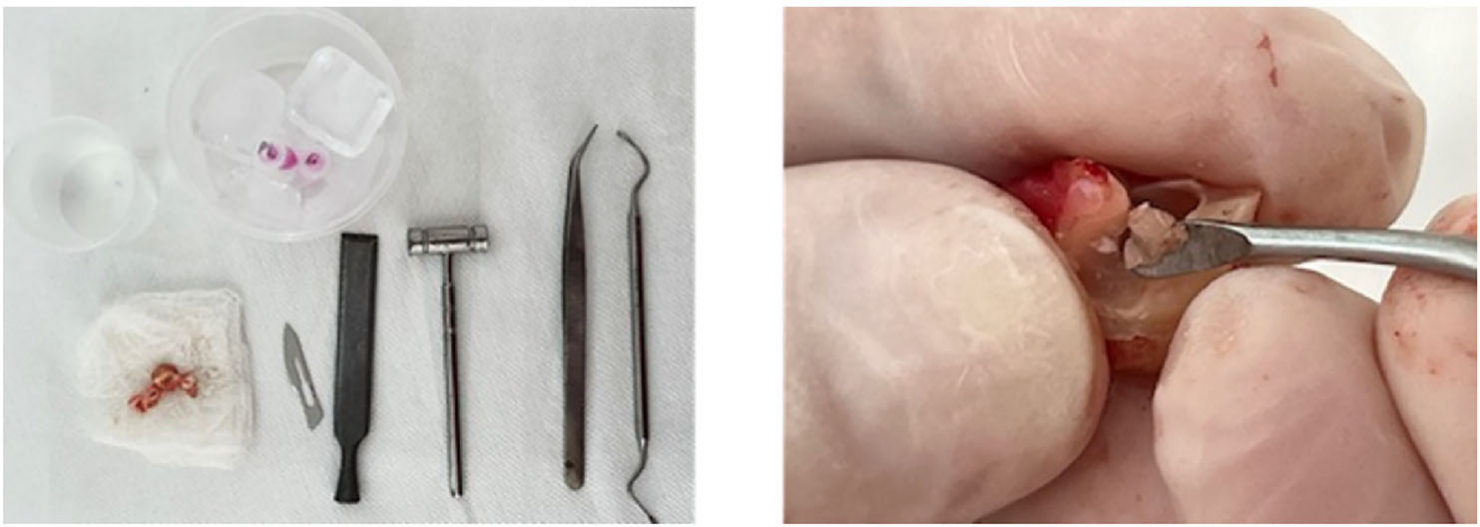
Tooth fracture and pulp collection. Fractured dental element and pulp tissue collected after harvesting the periodontal ligament.

**Figure 3:**
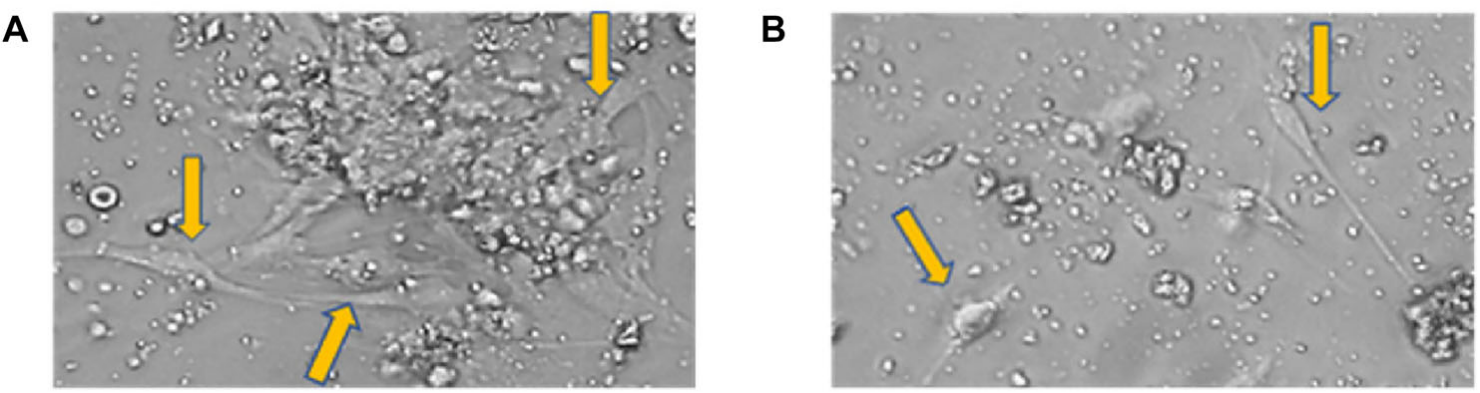
Third day of pulp and periodontal ligament isolation. Photomicrographs of pulp cells (**A**); periodontal ligament cells (**B**). Cells are indicated by orange arrows. 20× magnification.

**Figure 4:**
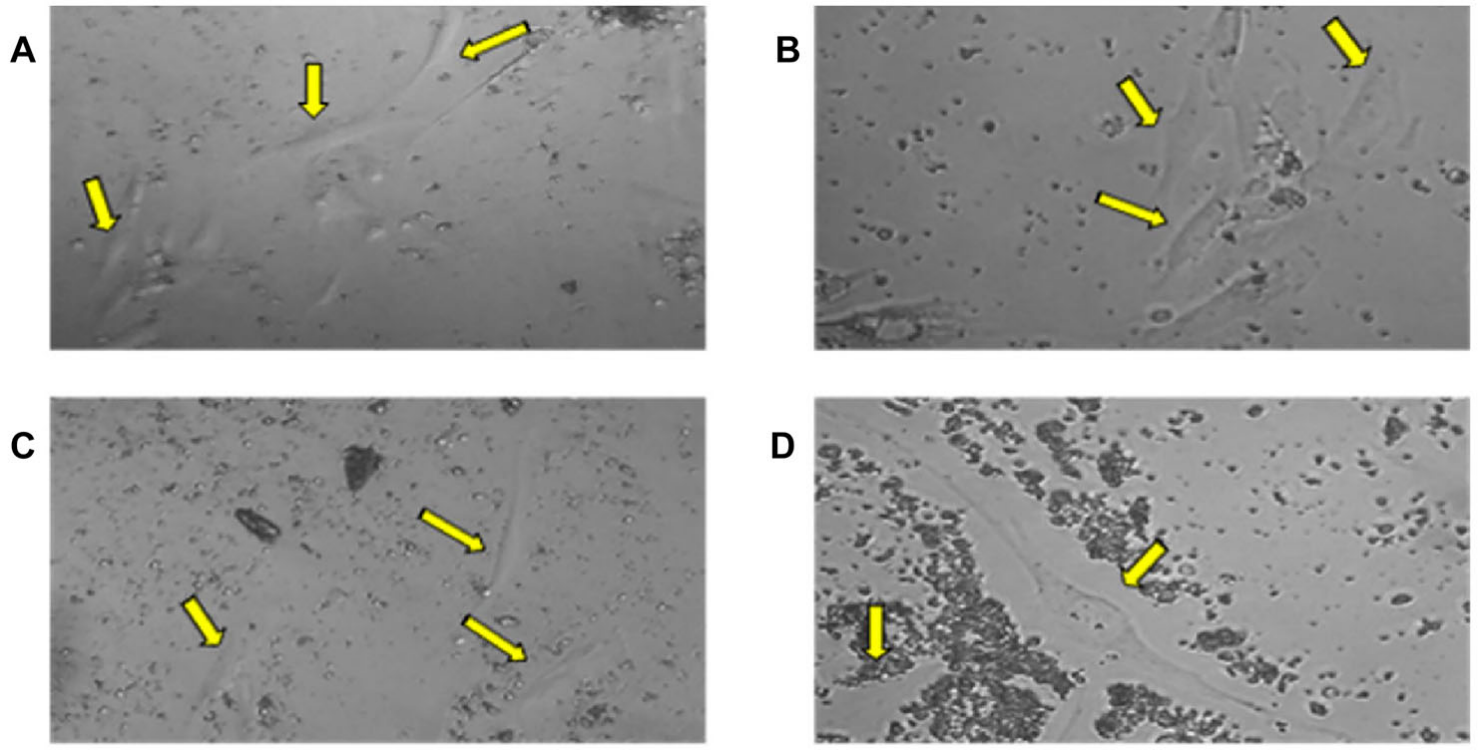
Sixth day of pulp and periodontal ligament isolation. Photomicrographs of isolated pulp (**A**‐**B**) and periodontal ligament (**C**‐**D**). Cells are indicated by yellow arrows. Images (**A**) and (**C**) are at 10× and (**B**) and (**D**) are at 20×.

**Figure 5:**
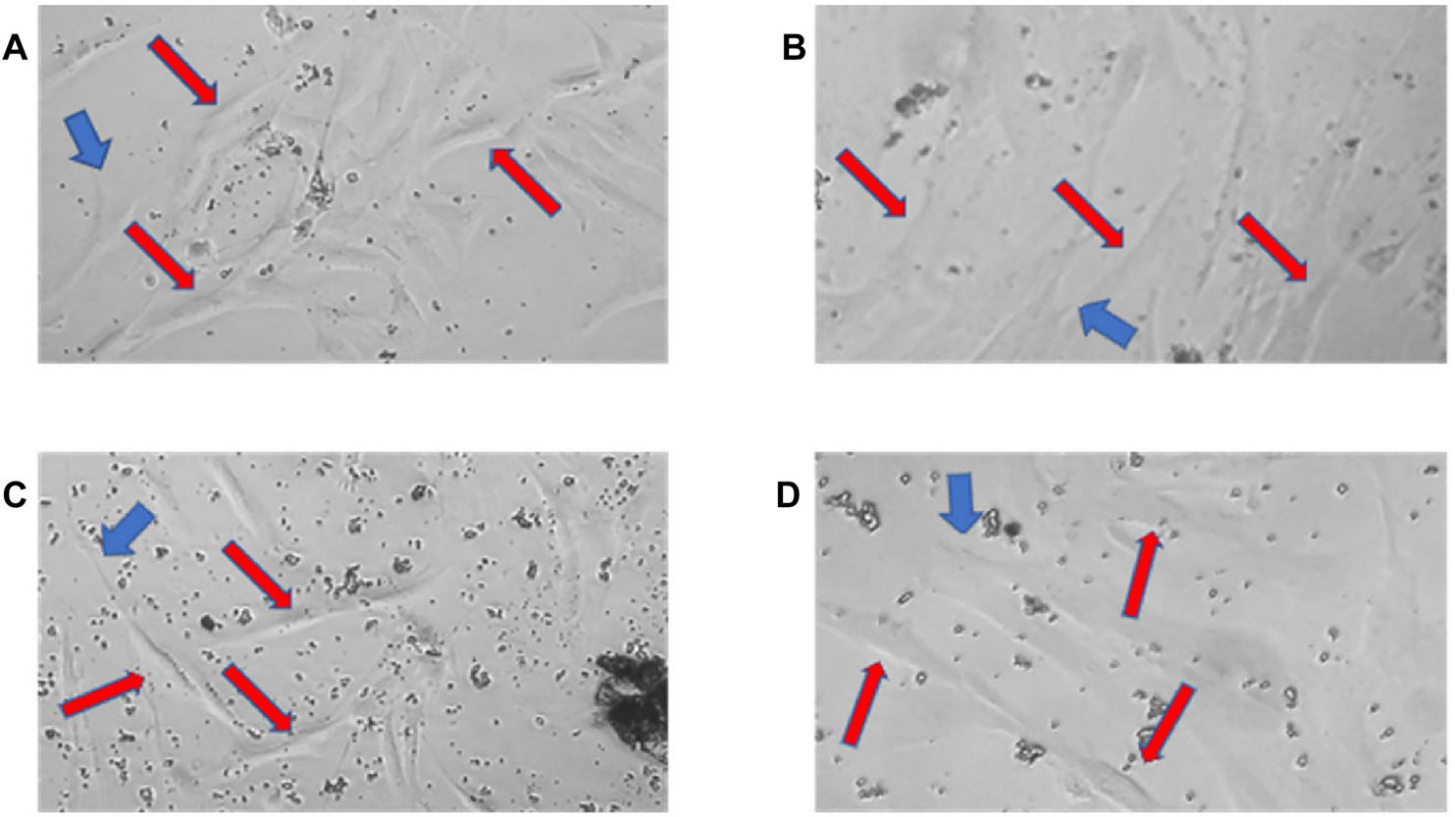
Ninth day of pulp and periodontal ligament isolation. Photomicrographs of the pulp (**A**‐**B**) and periodontal ligament (**C**‐**D**). The red and blue arrows respectively indicate the cells and the cellular extensions (used for cell migration). Images (**A**) and (**C**) are at 10× and (**B**) and (**D**) are at 20×.

**Figure 6:**
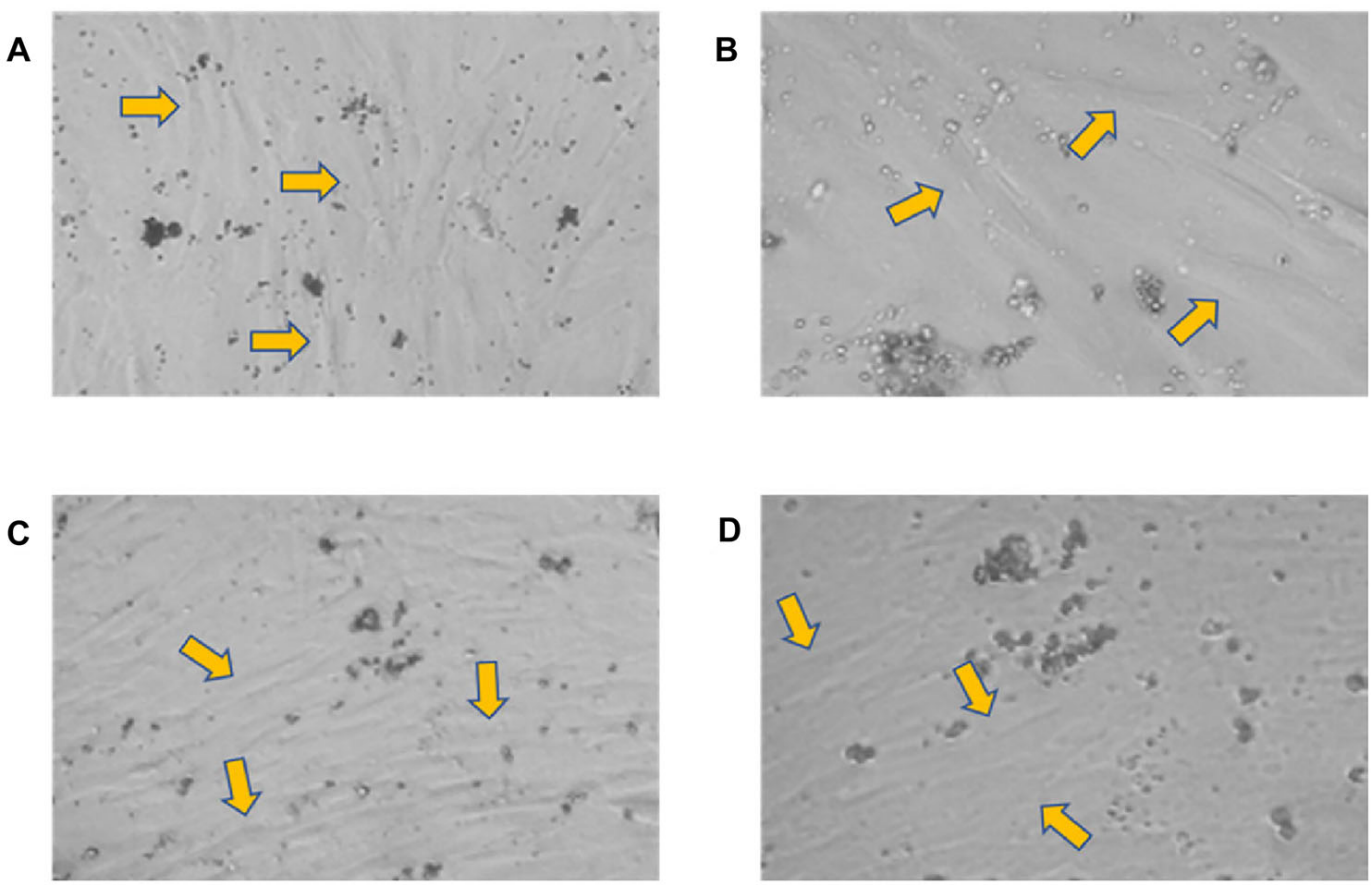
Twelfth day of pulp and periodontal ligament isolation. Photomicrographs of isolated pulp (**A**‐**B**) and periodontal ligament (**C**‐**D**). Cells are indicated by orange arrows. Images (**A**) and (**C**) are at 10× and (**B**) and (**D**) are at 20×.

Initially, the tissues collected by scraping the ligament in the roots and removing of the pulp from the pulp chamber after tooth fracture were placed in separate tubes, with SCM20% solution and kept on ice (Figs. 1 and 2). Subsequently, we proceeded with the cell isolation steps in the culture room. Upon the technique, we monitored the progression of cell growth using a microscope. For better cell observation, the images were cropped, displaying only part of the photograph. At this point, we observe significant changes, with cells being isolated every three days. On the third day, it was necessary to capture images of 20× instead of 10×. This is because observing the isolated cells, which are indicated by the orange arrows, is quite challenging at 10× magnification. It should be noted that the cells are located at the bottom of the plaque, attached and hidden beneath a significant accumulation of cellular debris and red blood cells (Fig. 3A‐B). On the sixth day of isolation, we detected an improved visibility of the cells at both 20× and 10× magnification (Fig. 4A‐D). The cells, albeit highly transparent, exhibited emerging extensions. On the ninth day, we began to observe the growth of cells, which were still very transparent. It was difficult to identify the cell nucleus at 10× and 20× magnifications, as indicated by the red arrows. We also notice the development of cellular functions, such as pseudopods, which are used for potential cell migration (Lampiasi, 2023), as shown by the blue arrows. Additionally, we observed a substantial reduction in cell debris due to complete washing with PBS (Fig. 5A‐D). On the twelfth day, there was a significant increase in the number of cells in both the pulp and ligament. The image taken just before the first trypsinization, shows some cells attached to the plate. These cells exhibit a fibroblastoid morphology, as indicated by the orange arrows (Fig. 6A‐D).

**Figure 7:**
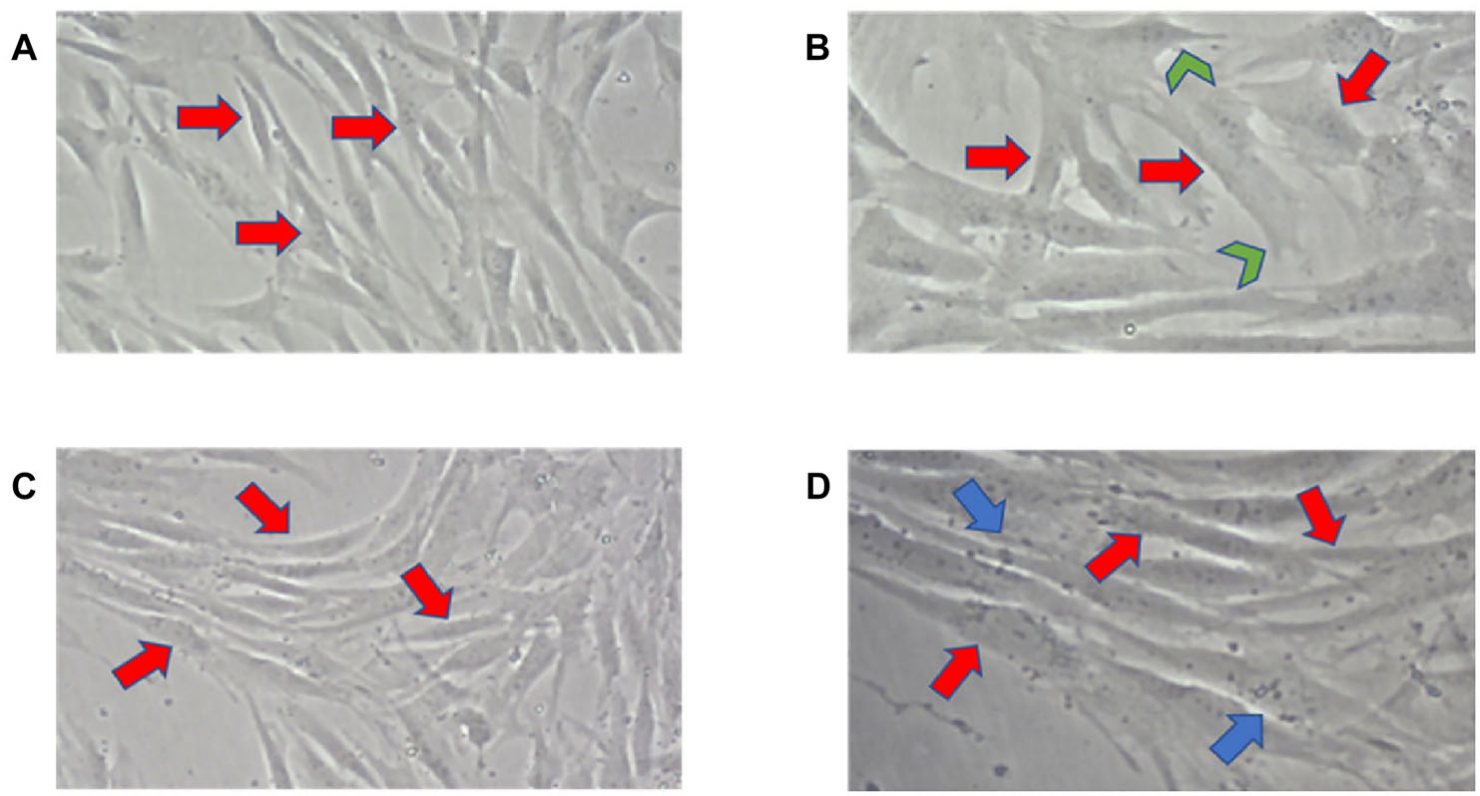
P0 passage of pulp and periodontal ligament cells. Photomicrographs from P0 passage of pulp (**A**‐**B**) and periodontal ligament (**C**‐**D**) Red, green and blue arrows respectively indicate cells, cellular extensions (filopodia), and cellular vesicles. Images (**A**) and (**C**) are at 10× and (**B**) and (**D**) are at 20×.

After the first trypsinization, the P0 passage allowed for a clearer observation of the cell culture´s growth of the cell culture, as indicated by the red arrows. Furthermore, the pulp cells (20×) exhibited diverse morphologies as a result of cell migration (Lampiasi, 2023; Schaks et al., 2019; Williams & Rousseau, 2022). This is supported by the presence of filopodia (indicated by the green arrow) (Gallop, 2020), which are expansions of the cytoplasmic membrane containing actin. These extensions undergo polymerization and form thin processes. At this stage, there was compelling evidence of cytoplasmic granules observed in periodontal cells (20×), as shown by blue arrows. These cells have a well‐developed secretory apparatus, containing cytoplasm that is abundant in mitochondria, rough endoplasmic reticulum, and Golgi complex. They release numerous molecules required for survival and growth and are likely involved in paracrine and endocrine functions (Trubiani et al., 2005) (Fig. 7A‐D).

**Figure 8:**
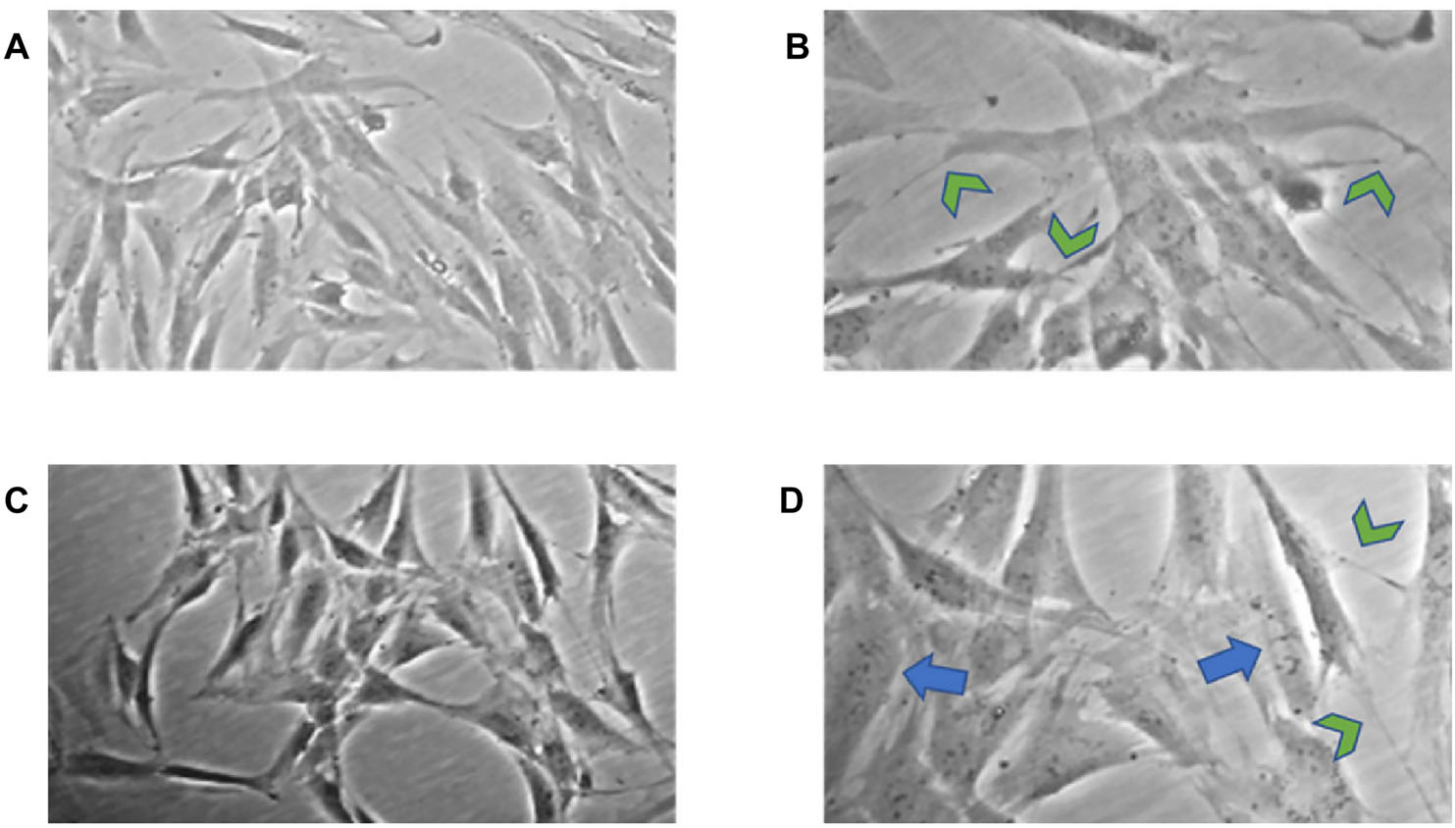
P1 passage of pulp and periodontal ligament cells. Photomicrographs from P1 passage of pulp (**A**‐**B**) and periodontal ligament (**C**‐**D**). Green and blue arrows indicate cellular processes (filopodia) and cellular vesicles, respectively. Images (**A**) and (**C**) are at 10× and (**B**) and (**D**) are at 20×.

The cells found in the P1 passage of pulp and ligament exhibit a high degree of similarity to those in the P0 passage. Our observations revealed the formation of numerous filopodia, as indicated by the green arrows, which represent the cell´s active search for contact with neighboring cells (Fig. 8A‐D). Although at this time, there is still no homogeneous culture of stem cells, due to the current passage, we can see specific features, such as fibroblastoid morphology, the formation of filopodia, and a high number of vesicles, which are indicative of stem cell characteristics.

**Figure 9:**
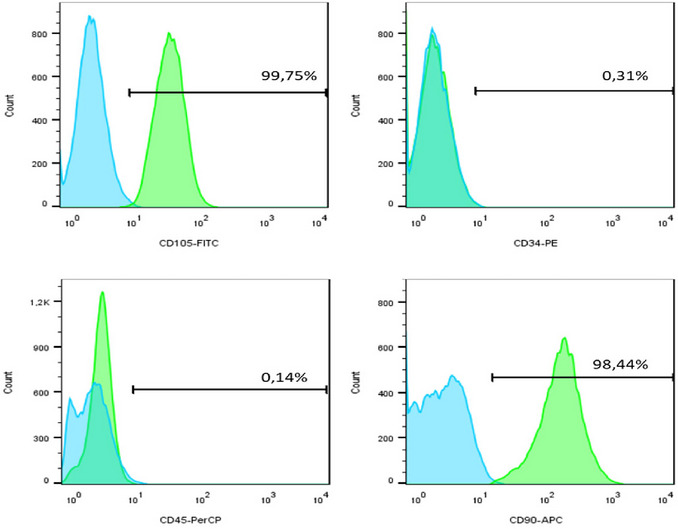
Expression of surface molecules in human dental pulp stem cells (HDPSCs). Graphs of the expression of CD105 FITC, CD34 PE, CD45 PerCP, and CD90 APC antibodies in pulp stem cells, demonstrating the percentages of expression.

**Figure 10:**
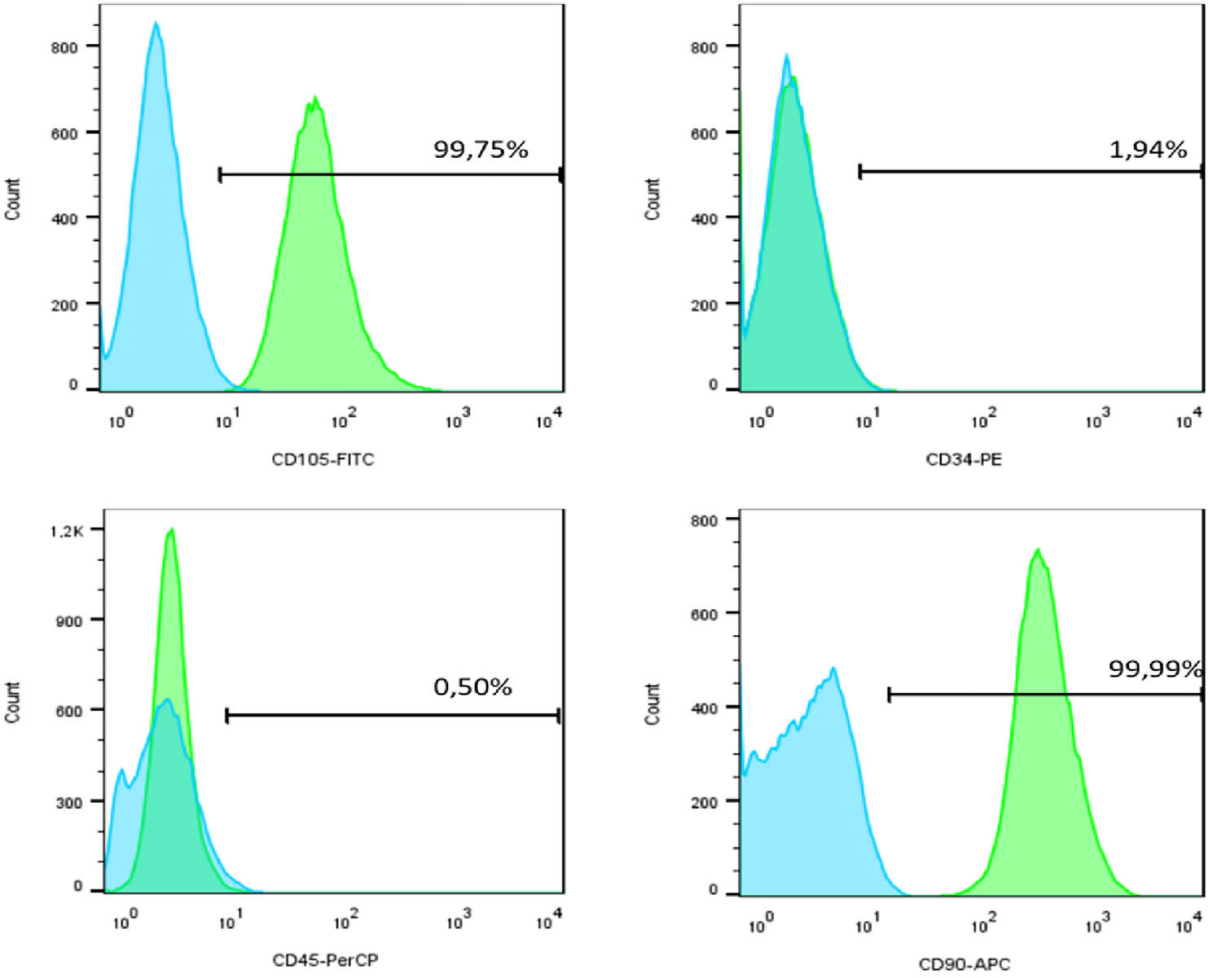
Expression of surface molecules in HPLSCs. The graphs depict the expression levels of CD105 FITC, CD34 PE, CD45 PerCP, and CD90 APC antibodies in periodontal ligament stem cells, showing the percentages of expression.

Following isolation and complete cell establishment, from the second to the third passage, the cells were submitted to cellular characterization (Figs. 9 and 10). As expected, the cells exhibited positive expression of the CD90 and CD105, while showing negative expression of CD34 and CD45. This confirmed their identity as stem cells.

**Figure 11:**
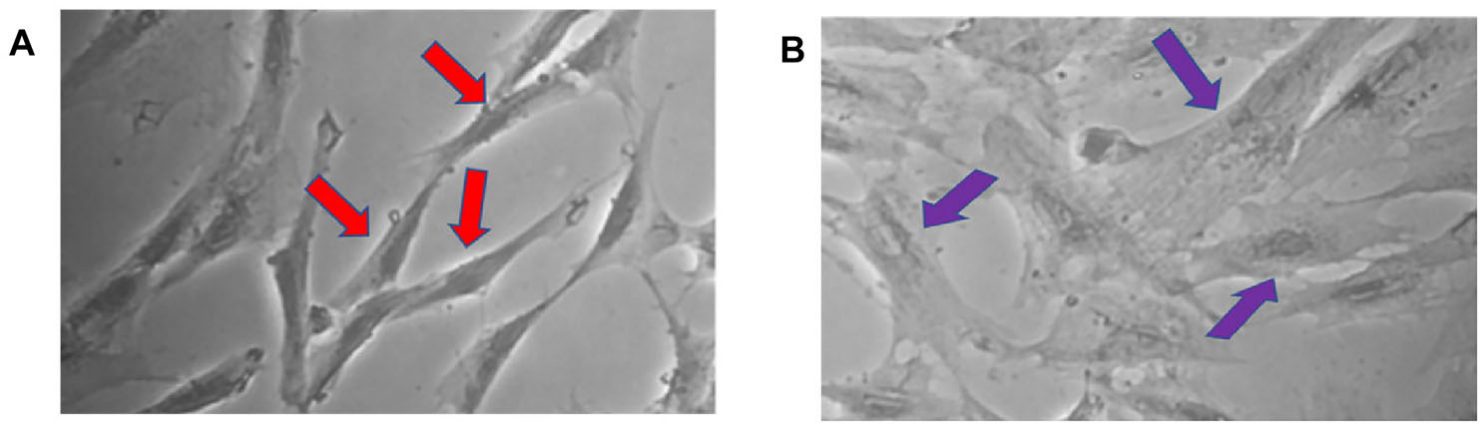
Stage of presenescent cells. (**A**) Negative control: cells that have not been exposed to hydrogen peroxide (H_2_O_2_) and have not undergone presenescence are indicated by red arrows; (**B**) cells that undergo presenescence when exposed to H_2_O_2_ are marked by purple arrows. Images are at 20× magnification.

The state of characterized cellular presenescence can be recognized by changes in cellular morphology. These alterations include enlargement, flattening (Petrova et al., 2016), and loss of uniformity in size and shape (Fig. 11).

### Time Considerations

Dental tissue collection: 20 min. Dental cell isolation: 2.5 to 3 hr. Cellular establishment until the first passage: 12 to 15 days. Cell characterization: plating, cell stabilization, and expansion, 3 days; cell culture room (trypsinization and cell counting), 1 hr; and incubation with antibodies, 30 min. Presenescence induction (50 hr): plating and cell stabilization, 24 hr; H_2_O_2_ treatment, 2 hr; cellular stabilization of the presenescent profile, 24 hr.

Note that the time required for cell acquisition and cytometry data analysis was not included because it can vary significantly depending on factors such as experimental design, cell type, choice of antibodies, and the operator´s expertise in using the cytometer.

### Conclusion

Although there are many methods for isolating, characterizing, and inducing the presenescence state, we observed the need for a study demonstrating in detail the importance and method of each step to obtain these cells. With these protocols, it is possible to obtain dental stem cells safely, effectively, and reproducibly.

### Author Contributions

**Kamila Sauer Veiga Leme**: Conceptualization; data curation; formal analysis; investigation; methodology; project administration; resources; validation; visualization; writing—original draft; writing—review and editing. **Márjorie de Assis Golim**: Conceptualization; formal analysis; methodology; software; supervision; validation; writing—original draft. **Aline Márcia Marques Braz**: Conceptualization; data curation; formal analysis; methodology; software; validation; writing—original draft. **Elenice Deffune**: Conceptualization; methodology; validation; visualization. **Daisy Maria Fávero Salvadori**: Conceptualization; funding acquisition; project administration; supervision; visualization; writing—original draft; writing—review and editing.

### Conflict of Interest

None of the authors have conflict of interests to declare.

## Supporting information


*This file contains a cell characterization table with the percentages of the expressions of the antibodies used for each pulp and ligament sample, and ethical approval*.

## Data Availability

The data that support this article are available in the Supporting Information section.
