# Supplementary material for: A Tutorial for Isolating, Characterizing, and Inducing Presenescence in Human Periodontal Ligament and Dental Pulp Stem Cells
Source: Curr Protoc. 2026 Apr 7;6(4):e70370. doi: 10.1002/cpz1.70370 (PMC13054634; doi:10.1002/cpz1.70370)
Supplement: Supplementary file 1 — This file contains a cell characterization table with the percentages of the expressions of the antibodies used for each pulp and ligament sample, and ethical approval. [file CPZ1-6-0-s001.docx]

**SUPPLEMENTAL INFORMATION**

A tutorial for isolating, characterizing, and inducing presenescence in human periodontal ligament and dental pulp stem cells.

Kamila Sauer Veiga Leme, Márjorie de Assis Golim, Aline Márcia Marques Braz, Elenice Deffune, Daisy Maria Fávero Salvadori.

**Summary of Supplemental Information**

Supplementary Table

1. **Table S1.** Cellular characterization.

**Ethical approval**

This study was approved by the Ethics Committee of the Faculty of Medicine of University of Botucatu (Unesp), (accept number: 5.081.033). Informed consent was obtained from all participants enrolled in the study.

**SUPPLEMENTARY TABLE**

1. **Table**

Table S1: Cellular characterizations.

| **Samples**  **Antibody** | **CD105 FITC** | **CD34 PE** | **CD45 PerCp** | **CD90 APC** |
| --- | --- | --- | --- | --- |
| 1. Pulp | 84.32% | 0.40% | 1.73% | 93.52% |
| 1. Pulp | 98.33% | 1.88% | 0.38% | 95.54% |
| 1. Pulp | 98.19% | 1.44% | 0.45% | 99.93% |
| 1. Pulp | 99.66% | 1.60% | 2.79% | 99.57% |
| 1. Pulp | 99.13% | 1.98% | 0.52% | 96.65% |
| 1. Pulp | 99.75% | 0.31% | 0.14% | 98.44% |
|  |  |  |  |  |
| 1. Periodontal Ligament | 97.86% | 2.22% | 0.58% | 99.71% |
| 1. Periodontal Ligament | 89.28% | 9.37% a | 1.96% | 99.08% |
| 1. Periodontal Ligament | 99.22% | 1.97% | 1.74% | 99.63% |
| 1. Periodontal Ligament | 96.65% | 1.76% | 1.40% | 99.79% |
| 1. Periodontal Ligament | 99.17% | 3.45% | 0.08% | 99.72% |
| 1. Periodontal Ligament | 99.75% | 1.94% | 0.50% | 99.99% |

*CD34 PE titrated with 2.5ul. not yet adjusted for correct titration (1ul).
